# Navigating the shift in Bangladeshi host community’s perceptions towards the Rohingya refugees: a declining sympathy

**DOI:** 10.3389/fsoc.2024.1346011

**Published:** 2024-02-06

**Authors:** Palash Kamruzzaman, Bulbul Siddiqi, Kajal Ahmed

**Affiliations:** ^1^South Wales Business School, University of South Wales, Wales, United Kingdom; ^2^Department of Political Science and Sociology, North South University, Dhaka, Bangladesh; ^3^Independent Researcher, Cox’s Bazar, Bangladesh

**Keywords:** Rohingya, refugees, host community, sympathy, frustration, humanitarian aid, Bangladesh

## Abstract

Generosity and selflessness from the host community in Cox’s Bazar were deemed to be instrumental in supporting Rohingyas who sought refuge in Bangladesh in 2017. Thousands of Rohingyas had to flee from their own country to save lives due to state-supported military violence. Initially, Bangladeshi media and civil society were largely supportive of the Rohingyas. However, the initial sympathy later withered away and may have turned into frustration and hostility. Based on 39 in-depth interviews with hot community members and humanitarian professionals, this paper argues that protraction of the crisis, inability to access natural resources due to the refugee camps, some Rohingyas’ involvement in various unlawful activities, a perceived sense of neglect from the international community, and disruption in local labour market/trade affecting cost of living conditions for low-income people seem to have played important roles in creating widespread tensions between the host community and Rohingya refugees. We contend that findings of this study will add to the critical scholarship of humanitarian development in deepening the understanding of host and refugee communities’ relationships. This paper will also have a positive impact on future policies toward harmonious coexistence between host communities and displaced refugees and potential sustainable solutions to the crisis.

## 1 Introduction

This paper focuses on host community’s perception towards the forcibly displaced Rohingya refugees from Myanmar who are currently living in congested Cox’s Bazar camps in Bangladesh. According to the United Nations High Commissioner for Refugees (UNHCR) Global Trends in Forced Displacement—2021 report (UNHCR, 2022), the total number of Rohingyas refugees is nearly 1.2 million and as of May 2023, Bangladesh hosts 961,175 Rohingyas.^1^ By providing shelter to almost 90% of the displaced Rohingyas, Bangladesh is one of the top 10 countries that host the largest number of people displaced across borders (UNHCR, 2018). Bangladesh has received extensive compliments from all corners of the world for hosting the Rohingyas (Alam, 2021). The Bangladeshi host community deserves special attention because they were the first people to support the distressed and exhausted Rohingyas before the state, NGOs, and the international community stepped in to help them. We, the authors, observed a “festive-like mood” in “welcoming” the Rohingya refugees when community groups and political leaders went to Teknaf with truckloads of food and other goods during the 2017 Rohingya influx in Bangladesh. Ansar and Md. Khaled (2021) identify four potential causes for such welcome to the Rohingyas including (a) religious similarities between the refugees and the hosts; (b) historical linkage with the Rohingya refugees; (c) solidarity from the major political parties and lastly (d) Bangladesh’s historical experience as a refugee-producing country during the liberation war in 1971. This warm response was somewhat contrasting to the refugee scholarship (Benard, 1986; Chambers, 1986) that suggest resource-poor and demographically surplus population often show resentment toward the arrival of new refugees. However, there has been a significant change in this regard as recent development shows that local media are increasingly reporting increased instances of drug peddling, unlawful activities, prostitution, and factional conflicts. There are also frequent suggestions of potential presence of extremist groups, and negative socio-economic and environmental impact of hosting a large number of Rohingyas (see Kamruzzaman et al., 2024). Against this backdrop, this paper aims to investigate the underlying reasons what may have turned a sympathetic welcoming atmosphere into a conflicting relationship. While we are aware of studies that outline Rohingyas’ plights, the causes of the crisis, roles of the international community (see, for example, Holloway and Fan, 2018; Haar et al., 2019; Chowdhury, 2020; Kamruzzaman et al., 2022) there is hardly any study that focuses on the host community and its perceptions toward the Rohingyas. We have interviewed the host community members and Bangladeshi humanitarian professionals to obtain a broader perspective in discerning the host community’s point of view behind the apparent shift in their attitude towards the Rohingya refugees. While a durable solution is not on sight after 6 years since the latest episode of the crisis begun in 2017, it is prudent to investigate the relationships between the Rohingyas and the Bangladeshi host community in forecasting pitfalls and ways forward for cohesive and inclusive policies towards potential solutions in Bangladesh, regionally and globally, where refugees are treated with dignity and respect instead of hostility and resentment.

The remainder of this article is organised as follows. The next section reviews the exiting literature that focuses on the relationships between the host communities and the refugees. This is followed by an explanation of the methodology of this study. Then we present the research findings by drawing on data from interviews conducted with the host community members and humanitarian professionals in Cox’s Bazar, Bangladesh. The next section analyses the research findings, followed by a conclusion section that is based on the findings and critical discussions made in this paper.

## 2 Host communities and refugee relationships in extant humanitarian scholarship

This section, based on a review of contemporary humanitarian literature, provides an important contextual background for investigating prospective reasons behind Bangladeshi host community’s apparent changes in their perceptions towards the Rohingya refugees. In a global context, recent humanitarian narratives highlight that the needs of the host communities and their voices should be adequately reflected in coordinating refugee crises and building cognate policies. This is visible in various international frameworks. For example, the Global Compact on Refugees (GCR) was developed to protect the refugees (in protracted displacement) and the communities that host the refugees. As the compact recommends “relevant actors will, wherever possible, continue to develop and support consultative processes that enable refugees and host community members to assist in designing appropriate, accessible and inclusive responses” (United Nations, 2018: 14). The Refugee Coordination Guidance (UNHCR, 2019: 6) also emphasises on, among others, ensuring the participation of refugees and host communities in an age, gender and diversity-sensitive manner. The UNHCR (2013: 90) global report on hosting the world’s refugees also insists that unless the needs of all concerned are taken into consideration, competition for scarce resources, like water or grazing pastures, may lead to environmental degradation, create tensions between host and refugee communities and even fuel further displacement. While these highlight the role and significance of host communities in major global frameworks, in practice, however, they have been persistently underrepresented or excluded in policymaking discussions and negotiations (Kirisci, 2018).

The impact on host countries and the local communities in which large numbers of refugees reside can be enormous. This can compound economic challenges for the host countries, particularly in those countries that can ill-provide for their citizens (UNHCR, 2011; Martin et al., 2018). In Western high-income countries, it is becoming increasingly unpopular as can be commonly found in Western media reports. The topic can have an extraordinary impact on a given country where the issue (hosting refugees) may have played a significant role in large-scale political transformation, such as the United Kingdom’s exit from the European Union (commonly known as Brexit). The impact can also be visible in the rise of right-wing populist ideologies and political parties in the European political landscape. It is important to acknowledge that low-middle-income country contexts can be very different as recent literature evinces that those nations often experience poverty due to a lack of resources and higher levels of economic vulnerability. As several studies (e.g., Ruaudel and Morrison-Métois, 2017; Fajth et al., 2019; Salas-Ruiz et al., 2021) identify that the economic conditions of the most host communities are not necessarily better than those seeking refuge in those countries. Consequently, refugees often face limited economic opportunities in their new places and sizable refugee influxes can put additional burdens on the host communities (Ruaudel and Morrison-Métois, 2017). Different studies (e.g., Chatham House, 2015; Miller, 2018; Bjørkhaug, 2020) also suggest that the low-middle-income host countries often argue that refugees are a strain on local resources and land. This can overwhelm health facilities and schools; impact the environment; strain infrastructure such as roads and bridges; encourage corruption in distributing services; and place a weight on social and administrative services (Miller, 2018; Bjørkhaug, 2020). There are also concerns that refugees can take jobs from nationals making a negative imbalance in the employment status (Chatham House, 2015; Miller, 2018) and driving up large increases in the prices of non-aid food items and housing in host communities (Alix-Garcia and Saah, 2009). Such situations may lead to economic competition over scarce resources between host and refugee communities and cause increased social tensions within the society, that sometimes could lead the host governments restricting or tightening up their support to the refugees (Ruaudel and Morrison-Métois, 2017; Fajth et al., 2019).

Hosting refugees can also cause significant tensions and conflicts between the host communities and the refugee population. Such tension may occur due to increasing competition over natural resources or services accompanied by humanitarian agencies and others (Crisp, 2003; Loescher and Milner, 2005). When the vulnerabilities of the refugees are combined with such conflicts, this might contribute to crime, violence, human trafficking, drug peddling, theft, and prostitution (Jacobsen, 2002; Siddiqi, 2022). Moreover, as part of political activism (new or old) where refugees seek to use the host country as a base for mobilising and recruiting new members, they (the refugees) then carry the significant potential to destabilise the countries that shelter them (Zolberg et al., 1989; Miller, 2018). Furthermore, if the refugees have been involved in radical activities or militant ideologies, the members/fighters of such groups can use their refugee status as a disguise, and host countries can suffer heavily from spill-over violence (Lischer, 2005; Salehyan and Gleditsch, 2006).

However, some studies (such as Whitaker, 2002; Betts et al., 2014) challenge a generalised understanding that refugees are a burden to the host countries. To illustrate, Whitaker (2002), in the context of Tanzania, found that while refugees can put pressure on local infrastructure, environment, and resources, but they can also provide cheap labour, and could expand consumer markets. From their observations in Uganda, Betts et al. (2014) assert that refugees can be economic assets through their networks and are able to use or create technology at higher rates than the local population through internet and mobile phone usage. Another potentially positive dimension to the host communities could be that refugees often gain major international attention and attract international organisations that help to bring resources, technology and jobs to an otherwise poor or remote area (Harrell-Bond, 2002; Landau, 2008). While Whitaker (2002) insists that hosting a large number of refugees may justify increased foreign aid, Martin et al. (2018) forewarn that aid often creates a vicious circle that can deteriorate the situation further. On the one hand, aid could limit the process of becoming self-sufficient for the refugees and might also turn the host community reliant on external assistance. On the other hand, some host countries might use a narrative of threat and fear that hosting a large number of refugees can transform the host countries’ governmental practices, and the expectations citizens have of their elected officials (Landau, 2003). Such narratives can then be used for leveraging increased international aid and other opportunities. For example, Sahin Mencutek and Nashwan (2021) show that Jordan and Turkey have assertively used refugee hosting as an opportunity to negotiate with international institutions and donor countries, offering to alleviate the “crisis” or providing temporal protection in exchange for political and/or monetary payoffs. This is reminiscent of the argument of Chambers (1986) and Waters (1999) that the economic patterns of both refugees and hosts need to be treated with caution where some can benefit from the influx, and at the same time, others can become marginalised.

Bearing the above discussion in mind, we can now turn our attention to the Bangladeshi host community’s relationship with the Rohingya refugees. There are very few studies that offer in-depth analyses on how the host community feel about the Rohingyas and the crisis. This is clear that the host community in Bangladesh shared a higher level of solidarity to the distressed Rohingyas. For Ansar and Md. Khaled (2021) assert that solidarity with the plight of Rohingya refugees is partly embedded in the shared memory of refugee experiences of the Bangladeshi people and its political elites. As in 1971, around ten million Bangladeshis (formerly East Pakistan) fled to bordering India to save lives from the atrocities and violence of Pakistan military crackdown (Datta, 2012; Lewis, 2019; Ravi, 2021), a brutal reminder of Bangladesh’s liberation war. Nevertheless, Khuda (2020) shows that like other studies mentioned above, the Rohingyas have made profound economic (competition for resources and job opportunities nationally and locally), social (rise in population and childbirth, health, deteriorating law and order), and environmental impacts (deforestation, waste generation, increased risk of landslide, groundwater contamination, impact on wildlife). The Bangladesh refugee emergency factsheet (UNHCR, 2018) clearly suggests that the rapid increase in the refugee population has strained the local community resources, infrastructure and public services and affected the economy, particularly in Ukhiya and Teknaf sub-districts. While a large number of international organisations, along with many national NGOs, are currently offering various support activities for the displaced Rohingya refugees, it is important to emphasise that Bangladeshi local communities were the first to respond to the influx of Rohingya refugees in 2017 and provided the lifesaving assistance. The hospitality and welcome by the local Bangladeshis have been detailed in the study of Holloway and Fan (2018) where exhausted and humiliated Rohingyas can be heard insisting that (being supported by the local Bangladeshis) was the time in which they felt most dignified because their struggles were recognised and they were provided with food, water and shelter, even though the Bangladeshi host community members were not obligated to do so. As Holloway and Fan (2018) provide further details where a man insisted that “the people of Bangladesh did something we will never forget. They did not need to do this and never needed to, but they still did and did it because they cared. This made us feel like our emotions, struggles and dignity mattered to them” (*ibid*: 15).

Against this backdrop, the following sections of this paper delineate the host community’s perception of the Rohingya crisis. But, before that, a brief account of the research methodology would be useful.

## 3 Research method

Based on qualitative research and the authors’ professional experiences with the Rohingya crisis, this paper aimed to explore the perception of the host community and the local humanitarian professionals toward the Rohingya refugees. Between September 2020 and March 2021, a total of 39 in-depth interviews (IDIs) were conducted to understand the host community’s perception. 21 IDIs (11 male and 10 female) were convened among the host community members from neighbouring areas of the Rohingya camps. The perception of Bangladeshi humanitarian professionals was also crucial as they work closely with the Rohingyas. Although working for humanitarian organisations in professional capacity but from a broader perspective, they can be considered as part of the host community. Their views offer a unique opportunity to contrast and complement the views of the host community members. While we are aware of researchers’ and respondents’ positionality in terms of how these may impact research ethics and objectivity (Holmes, 2021), we considered that both the host community members and the Bangladeshi humanitarian professionals were well placed to shed important light on the subject matter. 18 interviews were conducted with the humanitarian professionals (8 males and 10 female).^2^ All respondents were selected based on their availability and convenience. Thus, we needed to rely heavily on the process of snowball sampling to find and select respondents for the interviews. All interviews were carried out in Ukhiya, Teknaf and Cox’s Bazar city areas. Interviews were transcribed into standard Bengali. Two local research assistants helped the process, especially with the interviews of the host community members who spoke in local dialect. Data analysis was carried out based on the emerging themes in Bengali transcriptions. In addition, the authors’ field observations also helped to generate a detailed understanding in this regard. In doing so, primary and secondary codes were developed, and indexing was used in identifying patterns and emergent themes in the data. The thematic analysis was iterative and reflexive (Braun and Clarke, 2019). The authors occupy different roles in academia and practice (the lead author is based in a Western country, namely the United Kingdom, while the second author is also in academia working in a Bangladeshi University and the final author is a humanitarian practitioner in Cox’s Bazar). Their roles and personalities offer a unique position to add in-depth yet reflexive insights to this study. Both academic authors have had first-hand experience of working with the Rohingya refugees while the lead author also has experience of working on other refugee crises such as in Jordan and Afghanistan. The final author who is presently working in the humanitarian development sector also has had direct experience of the Rohingya crisis and at the time of data collection was based in Cox’s Bazar. Their experience enabled this study to adopt an approach that is informed by theory as well as wider real-world relevance. The authors are native speakers of Bengali and have in-depth knowledge of local culture, norms, values and politics. The authors exercised a great deal of reflexivity based on their experience and knowledge in minimising potential biases and maintaining objectivity when speaking to interviewees. While we are aware of researchers’ and respondents’ positionality in terms of how these may impact research ethics and objectivity (Holmes, 2021), we are confident that the views and interpretations offered in this paper are coming from interlocutors who are well placed to shed important lights on the subject matter (Mathijssen et al., 2023). We followed the ethical guidelines of the Association of Social Anthropologists (ASA) in conducting this study, including data collection and data analysis. As such, to ensure respondents’ anonymity and confidentiality, no names are included in presenting empirical evidence in this paper. We have only included respondents’ codes that we used to ensure their anonymity along with their occupation and gender.

## 4 Findings

Empirical evidence presented in this paper shows that Bangladeshi host community’s (henceforth host community) initial sympathetic attitudes towards the Rohingya refugees have already started to decline. Besides, an increasingly widening gap in trust between the host and Rohingya communities has created further dissatisfaction and tension. This study has identified several reasons for such dissatisfaction. Among others, the longevity of the crisis; unlawful activities of some Rohingyas resulting in perceived security risk; loss of land/forest and livelihood opportunities in setting up the Rohingya camps; disparity in humanitarian and other supports; increased living costs due to cheap labour of Rohingyas and cognate issues; price hike in house rent and everyday essentials were common themes for both the host community members and humanitarian professionals. The following sections will denote these issues based on the data collected in this research. The findings will be divided into two parts: the first part will show the perception of the host community, and the second part will discuss the perspective and perception of the humanitarian professionals.

### 4.1 Host community perspectives

#### 4.1.1 A declining sympathy

The host community members were very sympathetic and welcoming to the displaced Rohingyas at the beginning of the latest influx in 2017 (see Section 1). They viewed the Rohingyas’ vulnerabilities from a humanistic perspective. Many ordinary people from the host community came forward to help the Rohingyas in any ways they could. Various media published news on how the local community shared food and provided the Rohingyas with shelter. In this context, after 3 years of the 2017 Rohingya influx,^3^ this study aimed to explore the host community’s perception in discerning whether there has been any change in their comprehension towards the Rohingyas. Empirical evidence suggests that the host community members fondly reminisce the support they provided for the Rohingyas during the early days. For example, one respondent explained why the host community extended their hands to support the Rohingyas in 2017:

They [the Rohingyas] were kicked out from their country. They were tortured physically and mentally in Myanmar by their neighbour and the military. That is why the local people [of Cox’s Bazar] came forward to help them. (Respondent 5, female, community worker)

Some felt that as the Rohingyas are Muslim people, supporting and welcoming them was necessary to demonstrate Bangladeshi people’s (who are largely Muslims) solidarity. In addition to the religious sense of solidarity, they were also touched by the sufferings of the Rohingyas, as one respondent elaborated:

We treated them from a humanistic perspective as they [the Rohingyas] are also Muslim. Rohingyas are like our Muslim brothers and sisters. They have suffered a lot, and when they arrived in Bangladesh, their condition was not very good. I have a soft corner for them. (Respondent 8, female, housewife)

**Figure 1:**
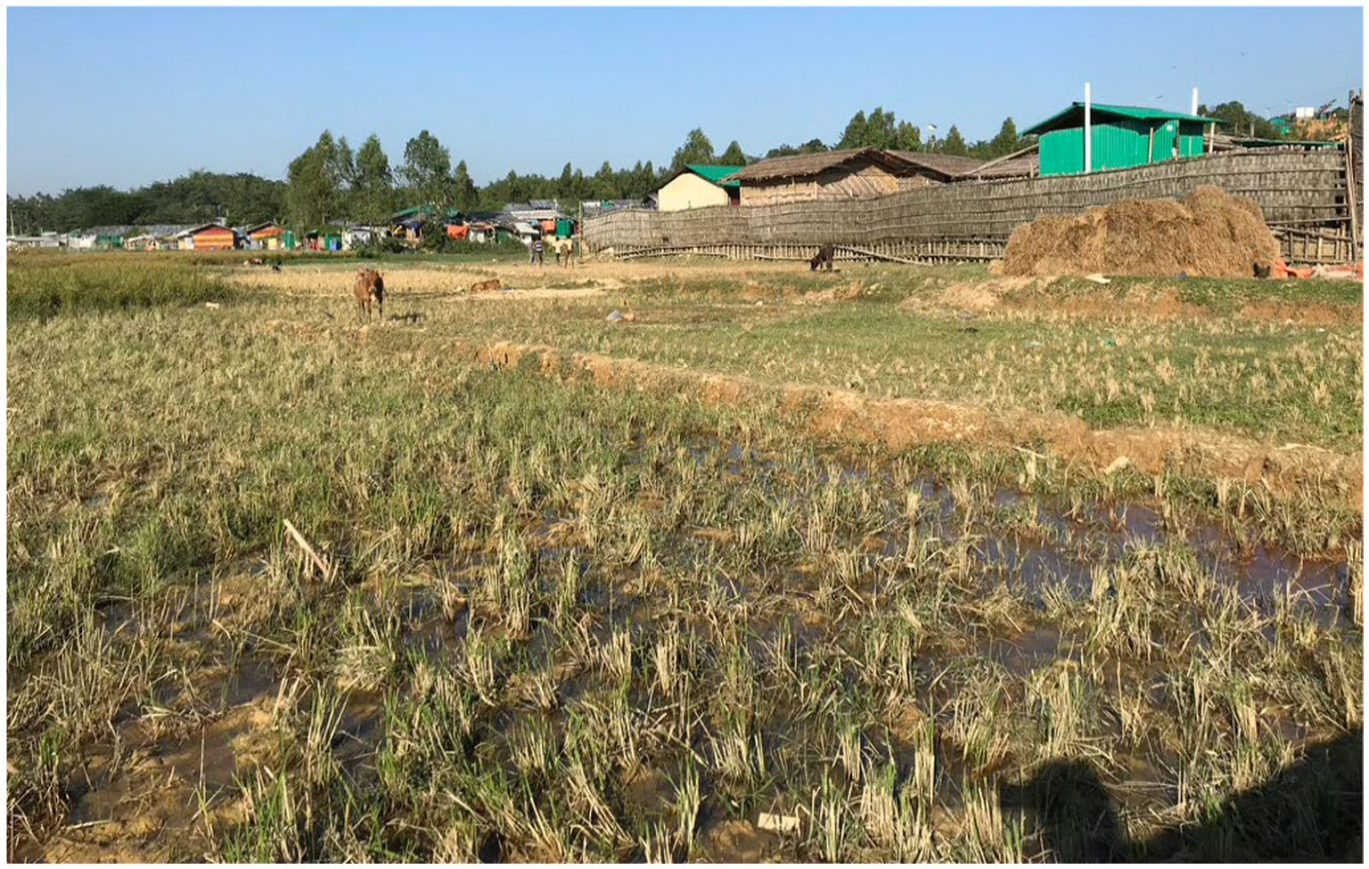
Boundary of Rohingya camps through the farmland of host community (source: authors).

Most respondents were in some agreement with the above as many from the host community helped the Rohingyas in various ways (e.g., by giving them clothing, food, shelter and helping the older people to move to a safer place). Both communities started to live in a peaceful environment while many from the host community allowed their lands for preparing makeshift camps or had to share the same forest for foraging and other aspects of livelihood (see Figure 1). A 31-year-old housewife (respondent 8) also stated that, “Although I am very poor and did not have much financial ability, I allowed my land for sheltering the Rohingyas”. Similar examples can be commonly found where the host community members sacrificed their lands and properties for setting up the Rohingya camps.

However, these warm perceptions about the Rohingyas have started to fade over time. Key to such change was the length of Rohingyas staying in Bangladesh and no sight of them returning to their homeland. Most respondents expressed their frustration about the duration of the crisis and Rohingyas staying in Bangladesh as a result. Members of the host community supported the Rohingyas and thought that it would be a temporary help and the Rohingyas would go back to Myanmar soon. But failure in repatriating the Rohingyas has created significant frustration among the host community. Such frustration may have transformed into anger and hatred, at least to some extent, threatening the peaceful coexistence between the host community and Rohingyas. As one respondent mentioned:

It has been more than three years^4^ since the Rohingyas took refuge in Bangladesh. You may know that if people from another country stay like the Rohingyas, it could create problems. The consequences would not be good for future if the Rohingyas stay in Bangladesh for a prolonged time. This is why it has already created anger and hatred among the host community. (Respondent 1, male, community worker)

While the longevity of the crisis (that Rohingyas staying in their community far longer than anticipated) fuelled their frustrations, the respondents also referred to other reasons for the change in their perception towards the Rohingyas. For example, unlawful and/or suspicious activities^5^ of some of the Rohingyas, impact on the environment, security issues, price hike of daily goods and the impact on the labour market were mentioned frequently.

Most respondents perceived that unlawful/suspicious activities have increased in their community. One host community member (who is also a community leader) raised her concern about the involvement of some Rohingyas in drug peddling/trafficking. Many people were particularly concerned about the increased connection of the Rohingyas in drug trade and trafficking. Cox’s Bazar is already known as a route of drug trafficking. The situation has aggravated further due to the increased involvement of the Rohingyas. One respondent shared that *yaba*^6^ has been a huge problem since the Rohingya influx in Bangladesh in 2017. The host community members were not happy seeing many Rohingyas involved in such activities. Some local newspapers have repeatedly reported such incidents that also furthered negative perceptions toward the Rohingyas. There is a growing fear of spreading such activities among the youth of the host community, as another respondent expressed:

The Rohingya crisis creates a harmful impact on the local population as many Rohingyas are involved in different evil deeds. Among them, various types of drug trade like *yaba*, hijacking, stealing, and prostitution are the most objectionable activities that negatively impact us as a community. (Respondent 9, female, Union Parishad Member)

Violent conflicts and clashes between the host community and Rohingyas are also a matter of deep concern. Extrajudicial killing and violent conflict between law enforcement agencies and the Rohingyas have become a regular occurrence in surrounding camp areas. Some respondents mentioned that many Rohingyas lure people from the host community to engage in factional violence and similar activities. This was suggested that some local people have died due to violent conflicts with the Rohingyas. A male respondent shared:

The problems in our area have been increasing since the Rohingyas entered Bangladesh in 2017. We have noticed a number of incidents of violence caused by the Rohingyas. They are also involved in violent conflict with the host communities, which is not good for either community. (Respondent 7, male, private service)

Violent clashes, drug trade, factional killing, and other illegal activities by the Rohingyas, as perceived by most respondents, have also created a fear about security risk in the host community. A small business owner explained that:

Many Rohingyas are getting involved in various types of radical activities, which is dangerous for us. People from the host community are feeling threatened. […] if this continues, I think there is a possibility of frequent conflicts between the Rohingyas and host community, which would be a huge security issue for the country. (Respondent 2, male, small Business owner)

Host community members also raised concerns about the impact on the environment due to deforestation in the hilly areas and environmental pollution in the region. Statement of a community worker from Ukhiya is relevant here, who mentioned:

I feel frustrated as we had to build refugee camps by cutting trees on the hills and forest areas. It is causing the rise of temperature and increasing various types of pollution in the area, which is impacting both host community and Rohingyas living in Ukhiya and Teknaf. (Respondent 11, male, community worker)

Some respondents also suggested that Ukhiya and Teknaf (two busiest sub-districts of Cox’s Bazar) have become a hub for many humanitarian professionals, which has a diverse impact on the local economy. The Rohingya crisis has increased opportunity for some people from the host community who might be intermittently working at various national NGOs as data collectors or as translators. Therefore, it created opportunities for some local people while overall livelihood expenses have gone up. But the people working as day labourers (and others in similar jobs) are suffering now due to the increasing numbers of Rohingya day labourers in the host community. A day labourer stated his frustration over the reduced wage for his labour:

We have been suffering after the Rohingyas came in our area. We used to get 500 BDT every day as our wage, but now many Rohingyas do the same job for 200 BDT. Thus, it impacts the local labour market as many Rohingyas have started working outside the camps. This is causing financial difficulties for us. (Respondent 17, male, day labourer)

The people from the host community are particularly concerned about the fear of being outnumbered by the Rohingyas. There is this tension that host community become marginalised as the number of Rohingyas is higher than the total population of Ukhiya and Tekanf. As one member from the host community shared his frustration by saying that “*Ukiyar manush bish khai mori zaileyo gom oibo*”, which means that it would be better for the host community people [in Ukhiya] to commit suicide than living with the Rohingya (Respondent 21, male, schoolteacher).

### 4.2 Perspectives from the local humanitarian professionals

#### 4.2.1 Changing circumstances for the local community

As many Bangladeshi staff are working in humanitarian organisations that are involved in providing humanitarian assistance for the Rohingyas, this study also aimed to glean their views on the Rohingyas to obtain a fuller perspective. These respondents are closely working with both the Rohingyas and host community of Cox’s Bazar. Like the host community members, all humanitarian professionals (henceforth humanitarian professionals) also believed that Bangladesh’s decision to extend its hands towards the Rohingya refugees was the right thing to do. For them, it was a decision to save the lives of the Rohingyas.^7^ The justification for helping the Rohingyas by providing shelter, and other facilities was coherent among humanitarian professionals and the people from the host community. As one staff of a national NGO mentioned:

It was not their choice to enter Bangladesh; instead, they were displaced by force and brutality. So, it was an appropriate decision by the Government of Bangladesh to allow the Rohingyas to take refuge in this country. (Respondent 22, female, NGO worker)

All humanitarian professionals think that although the host community members were the first responders to the distressed Rohingyas in 2017, but they were drifting away from early warm feelings. According to some humanitarian professionals, the host community of Cox’s Bazaar depict a unique case where an initial sympathetic feeling has turned into a wrath (*Odor boro Guish’sha*) towards the Rohingyas as the crisis got protracted and for other reasons detailed below.

Some humanitarian professionals felt that there was a growing sense of feeling among the host community of being outnumbered and becoming the “new minority”. This observation was made in comparison to around one million Rohingya refugees living in the area that the host community consider as their own territory. Rohingyas have caused significant constraints to their livelihoods, especially for the people who were highly dependent on the natural resources of reserve forests that have been allocated for Rohingya camps. As one respondent from an international NGO mentioned in this context:

Many locals had to abandon their houses and land that are now inside the camps. These used to be their only possession for survival and subsistence. After the settlement of Rohingyas in the camps, when they (locals) tried to access or use these lands again, they were regularly countered by the Rohingyas who are very compact as a group. People they once welcomed (Rohingyas) are now quite aggressive towards them (the local community). Often such encounters lead to violent clashes. (Respondent 23, female, international NGO worker)

**Figure 2:**
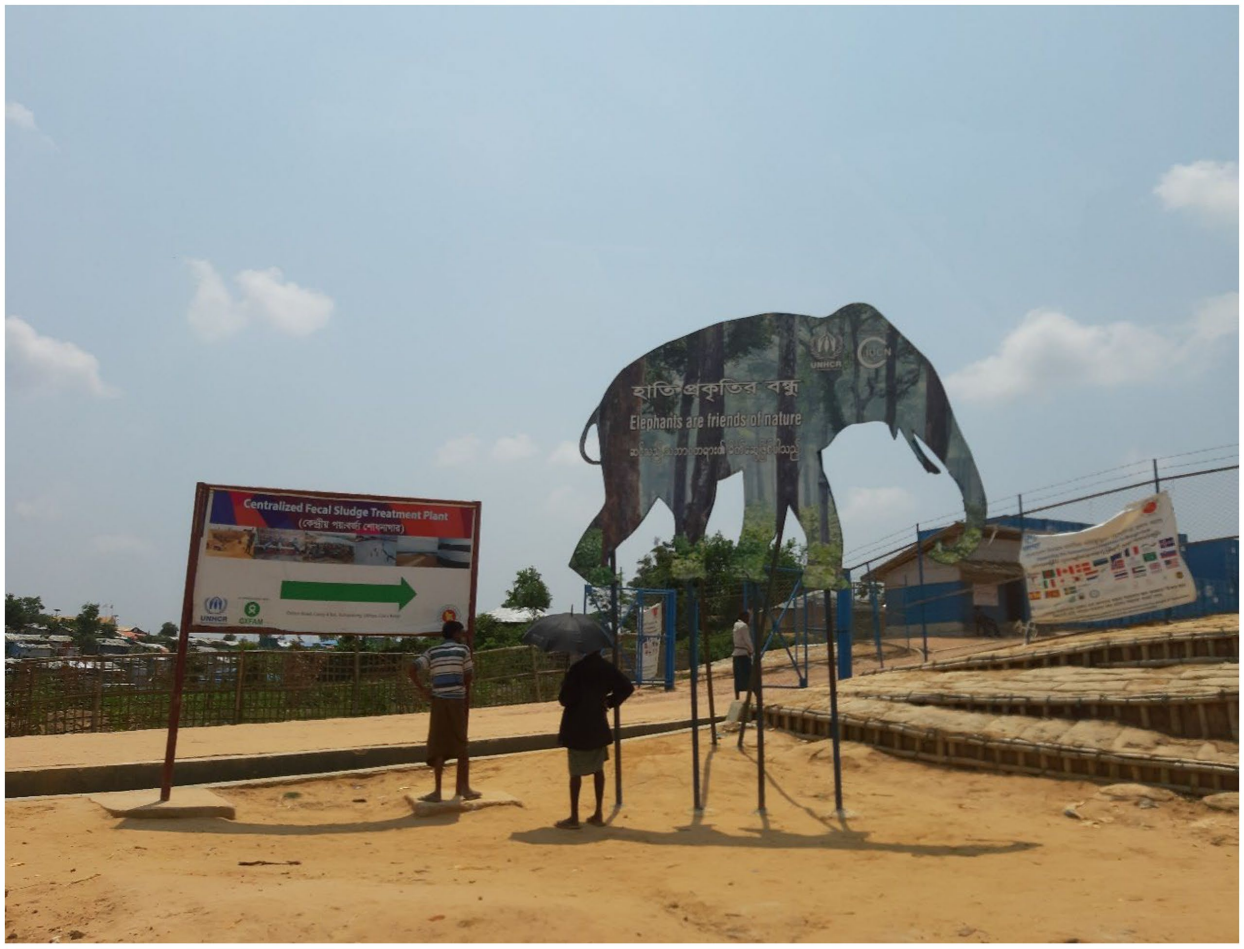
A cut out of an Elephant in front of Rohingya camps (source: authors).

Moreover, for some respondents, the environmental and ecological impact have contributed to the host community’s change of perception. The camps have been built on lands that were mainly reserve forests and now they see that forest is being destroyed. The natural habitat of many animals was destroyed as many camps were built on the hills and forest areas that were safe zone for wildlife including the elephants (see Figure 2). Moreover, the use of groundwater has increased, lowering the level of groundwater significantly.

**Figure 3:**
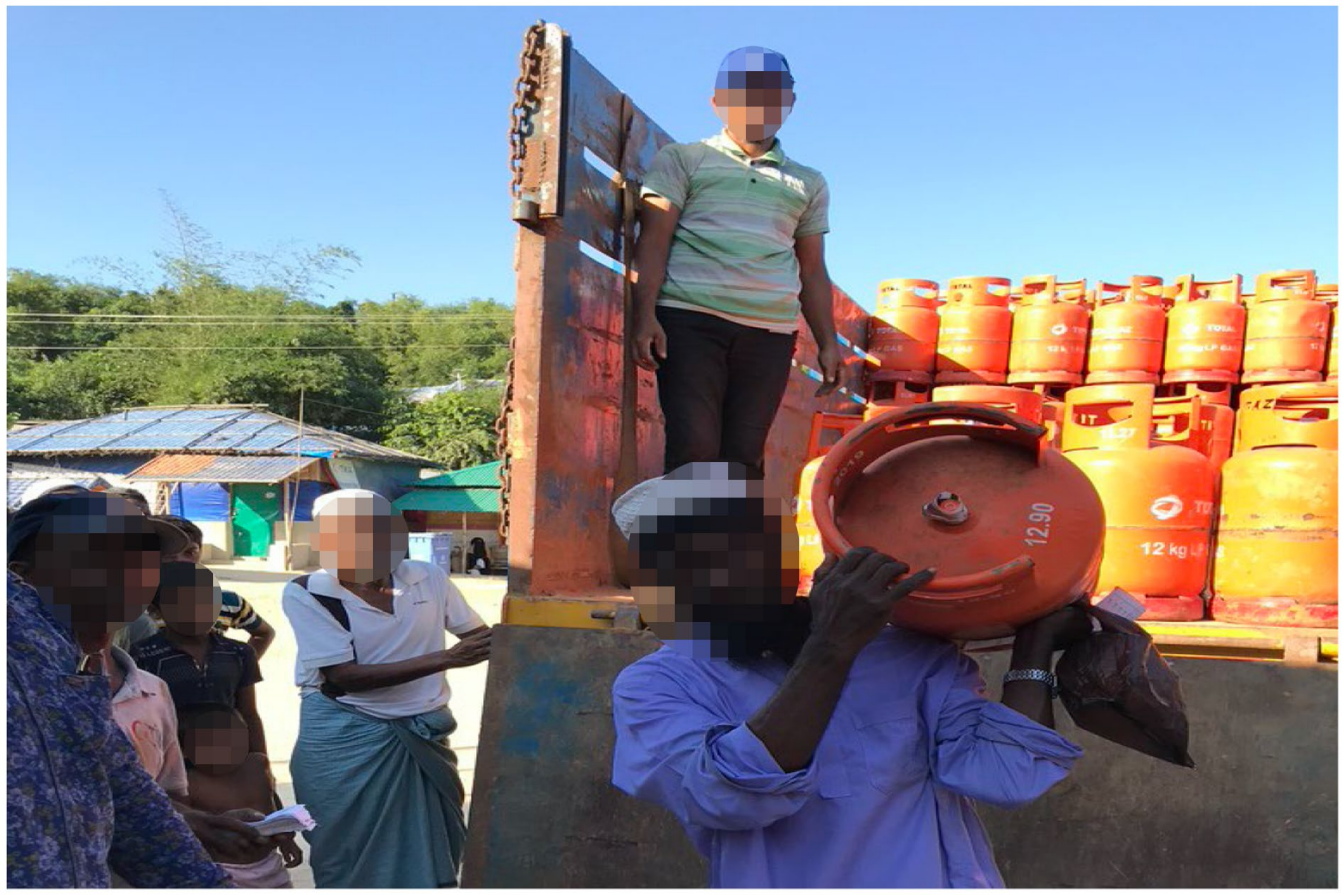
Rohingyas receiving monthly supplies of gas (LNG) cylinders (source: authors).

Cox’s Bazar district is one of the poorest areas in Bangladesh. However, the massive aid operation to provide basic needs and humanitarian assistance for the Rohingyas has negatively impacted the psychology of host community members. They *feel* that millions of dollars are being spent on aid for these refugees (see Figure 3), while the host community suffers from a lack of economic and employment opportunities. This has been described as one of the main reasons for frustration among the local community.

**Figure 4:**
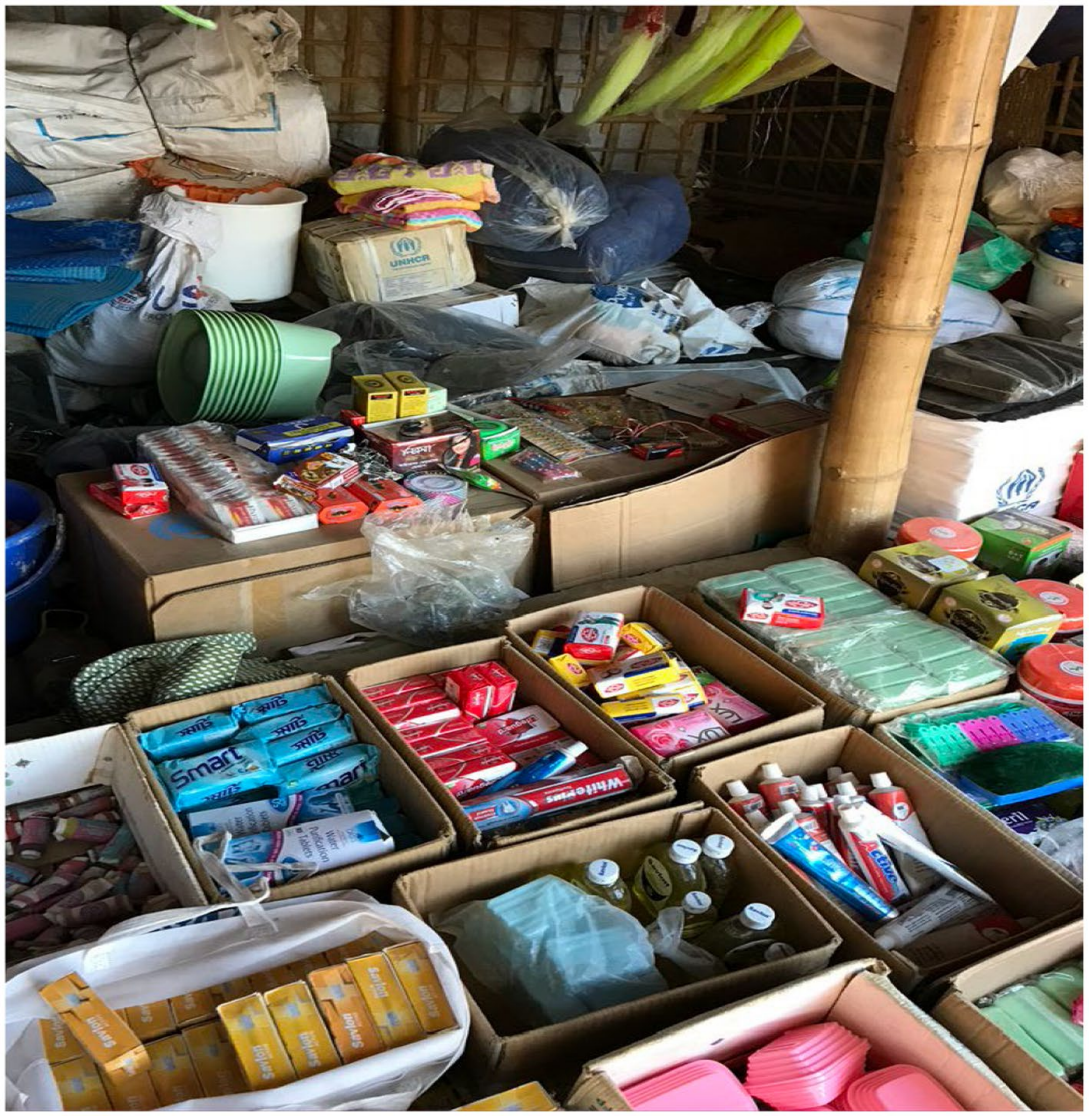
Products available in “surplus relief market” (source: authors).

Aid *surplus* is another issue that is disrupting the local market system, impacting local traders and individuals. Many relief items are being sold in local markets around the camps by the Rohingyas to earn some cash. There are regular consumers from the local communities for these commodities (both food and non-food items). Local customers buy these commodities from the “Rohingya markets” instead of local shops. Such a market is often termed a “surplus relief market” or “*Rilifor malsamana besede Bazar*” in the local dialect (see Figure 4). This has primarily impacted small and medium shopkeepers as they face customer shortages and are bound to reduce the size of their businesses. A humanitarian professional working for an international NGO mentioned in this context:

Customers continuously seek commodities at lower prices. If they can have soap, a pack of detergent, or a bottle of oil 20%-40% cheaper than local shops, why would they not buy it? So, customers might be gaining, but the small shop owners are losing their livelihood. (Respondent 25, female, international NGO worker)

Furthermore, the Rohingya crisis seemed to have impacted the mobility of host community members. To illustrate, Rohingya mobility is restricted within camps and officially, they are not allowed to go outside the camps without prior approval from the camp management authority. There are government protocols and check-posts to restrict the Rohingyas’ mobility. As a result of this, host community members are frequently stopped and asked to show their proof of nationality and identity, even for their random movements within neighbouring areas of the camps. Moreover, accessing government services for official credentials like birth registration of children and issuing National ID cards and passports for Bangladeshi nationals has become difficult. This is because the GoB made it stricter as several Rohingyas are trying to avail of these services to flee from the camps. In this context, a humanitarian professional from an international NGO mentioned:

Rohingyas have restrictions on their daily physical mobility as they cannot go outside the camps. The camps have fencing in most parts to control their physical mobility. Humanitarian organisations have a huge objection to this policy initiated by the Government. (Respondent 24, male, international NGO worker)

Also, regardless of restrictions to remain inside the camps, many Rohingyas frequently travel outside of the camps for their search for additional income as daily labourers (mainly). They are selling their labour in a much cheaper rate than the existing local wage rate, reducing the scope of employment for local people dependent on daily labour for survival. One humanitarian professional mentioned in this context:

Rohingyas are regularly receiving relief from different organisations. So, it is easy and convenient for them to work at a cheaper rate. But for local day labourers, the situation is quite the opposite. They primarily work on a daily wage basis, which is insufficient to ensure their subsistence. Therefore, it is hard for them to compete with the Rohingyas, and people are hiring Rohingyas as their service is cheaper and local labourers are out of jobs. Many Rohingyas are engaged in fishing and dry fish processing, which also squeezes the employment options for the locals. (Respondent 24, male, international NGO worker)

Furthermore, the Rohingya influx has also increased the scope of work for a number of humanitarian organisations. This means thousands of their workers or staff now live near/around the host community for their day-to-day jobs with the Rohingyas and the local people. They are renting properties/houses for setting up offices as well as for staff accommodation. Consequently, there has been a sudden yet unprecedented spike in house prices/rent,^8^ and daily supplies of fresh foods. A humanitarian professional from an international NGO mentioned the following in this context:

House owners are keener to rent out their properties to humanitarian organisations or staff as they are bound to stay here for their work, and somehow, they are managing it. It might be manageable to rent a house or office at a double rate for these staff as they get higher salaries or have organisational backup. But suddenly, it became unmanageable for the other people who lived in these areas as tenants with lower incomes. (Respondent 26, male, international NGO worker)

Like the host community respondents, humanitarian professionals also perceived that the engagement of Rohingyas in illegal or unlawful activities poses a great threat to the community and Bangladesh’s national interest. Cox’s Bazar is historically known for drug and other substance trafficking as it is close to the drug route—Golden Triangle. One of the most commonly trafficked drugs through this route to the country is *Yaba,* or madness drug—a combination of caffeine and methamphetamine. The Rohingya camps have become a new gateway or transit route for shipments of these drugs. In addition to drug peddling, petty thefts, mugging and similar instances are reported to have increased in the area. A humanitarian professional from an international NGO mentioned in this context that:

For the Rohingyas, this [drug peddling] is an option for easy money. Some of them are engaged in drug trafficking and gold smuggling. Apart from these, petty crimes like theft, mugging, snatching, etc. have also increased. Previously, there were very few incidents of theft or snatching. But now, these are frequent. Every night something is being stolen from the houses of the local community. It does not matter if it is a pair of shoes or something more valuable. Also, thousands of humanitarian workers live here and usually possess mobile phones, laptops, or other electronic items. Therefore, their houses are recurrent targets for theft or snatching by the Rohingyas. (Respondent 27, male, international NGO worker)

Respondents also raised that factional violence among the Rohingyas is evident in the neighbourhood. Several armed groups are operating and engaged in arms smuggling, making local arms, selling, extortions, kidnapping and collecting ransom, etc. in and outside the camp areas.

Bangladesh is now in a security risk due to the increased number of drug trading and smuggling among the Rohingya. Cox’s Bazar has become a route for drug trading. (Respondent 33, female, international NGO worker)

Violent clashes by the Rohingyas are mentioned to be frequent phenomenon for typical for establishing control or power over the territories/camps claiming innocent lives or executing targeted killings in the camps and the local community. This has created fear within the host community specially for them who are living adjacent to the Rohingya camps. People from the host community raised a similar concern, which is one of the key reasons for turning a sympathetic stance into hatred and causing significant tension between the host community and Rohingyas.

Lastly, this was also flagged up that many Rohingya women are forcefully deployed as sex workers and regularly shipped in and out of the camps to Cox’s Bazar. Many humanitarian workers claim that hotel-based sex work has increased in Cox’s Bazar. Cox’s Bazar, as a national and international tourist attraction has become a lucrative place for prostitution. A humanitarian professional from a national NGO mentioned:

The scope of prostitution has increased in the area due to the vulnerability of the Rohingya female. We even heard that Rohingya girls were kidnapped [potentially to be forcibly used for sex work in the city area]. (Respondent 32, female, NGO worker)

Increased prostitution is a concern, and the host community disapproves of it as it negatively affects their society and culture.

## 5 Discussion

Several studies suggest that Bangladesh’s history of taking refuge in neighbouring country during its liberation war in 1971 may have played a role (Datta, 2012; Lewis, 2019; Ravi, 2021) for the Government of Bangladesh to shelter the Rohingyas. Perhaps, this was true for political decision, but this study found a sense of shared values (based on religion and culture) and humanistic perspective were instrumental for Bangladeshi host community’s initial sympathetic response toward the Rohingyas. Respondents of this study warmly remembered the early days when they assisted traumatised Rohingyas to the best of their abilities and intentions. Although some degree of compassion towards the Rohingyas can still be found, but this study shows that a sympathetic undertone is gradually withering away. Empirical evidence presented above suggests a range of possible reasons in this regard. One of the main reasons is the unexpected length of the crisis. From the passionate notes of the respondents during the interviews, we feel that the Bangladeshi host community was happy to shelter and support hundreds and thousands of Rohingyas for a short period of time. They felt this was their religious or humane duty to stand by the persecuted Rohingyas. Protraction of the crisis seems to have stretched the generosity of the host community and contributed to declining their initial sympathy. This is reminiscent of Benard (1986) and Chambers’ (1986) observation about resource-poor community’s response towards the arrival (in this case long term stay) of large number of refugees.

Most stakeholders of this crisis, including the Rohingyas, intend to return to Myanmar, of course, in safety and in a dignified manner (Kamruzzaman, 2022; Mahmud, 2022). There have been many consultations and negotiations in national, regional and global forums. But, nothing has resulted in safe and secured voluntary repatriation of the Rohingyas at the time of writing this paper. We find that this has been a major cause of dissatisfaction (with no fault of the Rohingyas as a safe dignified repatriation to Myanmar is not in their hands). During our interviews with the host community members and humanitarian professionals, this was a recurring issue and we found that the Bangladeshi host community seems to have become tired of being a great host. Furthermore, a gradual declining sympathy was evident among the neighbouring host population of the Rohingya camps due to several other reasons. Among them, the most notable are unlawful and suspicious activities (including drug peddling/trade), factional violence, potential presence of extremist groups, petty theft and mugging, and a rise in prostitution in local areas. Similar to the arguments of Jacobsen (2002) and Fajth et al. (2019) about increased social tension due to rise in crime, human trafficking, drug peddling etc., some Rohingyas’ involvement in drug peddling/trafficking, along with other unlawful activities, was frequently mentioned by both the host community members and the humanitarian professionals. However, a rise in prostitution and the presence of extremist groups were not explicitly described by the respondents. Perhaps, it was not easy or comfortable for them to relate these issues with a group of people with who they perceive to have some connections/solidarity. While the issues such as a rise in prostitution and extremist activities came less strongly from our respondents some studies, however, suggest that to be the case (see, for example, Hosen, 2019; Khuda, 2020; Hossain et al., 2021). Furthermore, our respondents’ concern about the security risk is also shared in other studies highlighting that this can turn into a wider security threat to the region (Kipgen, 2019; Hossain et al., 2020; Ansar and Md. Khaled, 2021).

This article also suggests that the perceived loss of land and forest by the local people could be another important reason for the host community’s declining sympathy towards the Rohingyas. Many members of the host community, before the influx of the Rohingyas, were relying on the lands and forests that had to be given up or destroyed to shelter the Rohingyas. Interviews with the respondents, authors’ own observations (for example, see Figures 1, 2), and other studies (such as Hassan et al., 2018; Hasan et al., 2021) reconfirm this and augment the arguments of competition over scarce resources (Miller, 2018; Bjørkhaug, 2020). As mentioned above, the host community was perhaps willing to make short-time sacrifices but since the losses of lands and forests are significantly affecting their livelihood opportunities, they seem to be getting increasingly frustrated with the situation. Such feeling is further aggravated by other developments as daily jobs are becoming more competitive and wages might be even lower since the cash-strapped Rohingyas’ availability to do labour-intensive daily jobs, as observed by Alix-Garcia and Saah (2009) and Chatham House (2015). While income for many people in nearby areas has decreased, price hikes for everyday essentials and local house rent have created a major cost of living crisis for many people. This must be also acknowledged that the crisis has created job opportunities for humanitarian and other workers, but this study reveals that the host community feels this was mainly for the people from outside of Teknaf and Ukhiya along with many foreigners. This issue can also be related to the perceived disparity in aid and other support systems. Similar to the arguments of Landau (2008) and Harrell-Bond (2002), certainly, the Rohingya crisis has brought international aid to the community. The importance of humanitarian assistance and other cognate supports was clearly visible during our visits to the camps. Nevertheless, the evidence presented in this paper is more congruent with Martin et al. (2018)’s caution that aid might deteriorate the situation further as the host community feel that almost all the supports are available for the Rohingyas and there is very little for them. Humanitarian professionals also confirmed this although, when probed further, they were unable to provide any specific data/statistics in support of their claim. During our field trips, visibility of humanitarian support to the Rohingyas was clearer than the support available for the host community. The establishment and growth of surplus relief markets (“*Rilifor malsamana besede Bazar*”) are apparent markers of the perceived disparity. We do not think the feeling is generalisable but the responses that suggest local people feel like they have become a minority in their own land and committing suicide might be a better option (see above) can be symptomatic of the feelings of some sections of the host community. Moreover, the protraction of the crisis means that other new crises have emerged in the global context and the attention of the international community may have shifted elsewhere. As already found in other instances (Martin et al., 2018; Miller, 2018; Bjørkhaug, 2020) Bangladesh is also experiencing huge economic strain for hosting large number of Rohingyas. There is a large gap in funding for Bangladesh^9^ despite a recent major donor conference that aimed to generate “sustained support for the Rohingyas” (for more details, see Kamruzzaman, 2022). This is beyond the remit of this paper, but Levine and Saez’s (2022) argument that donors’ attention to the Ukraine crisis could mean less funding for other refugee crises in the world, including the Rohingya crisis. Based on the empirical evidence included in this paper, we presume this might accentuate the Bangladeshi host community’s perceptions of disparate aid support. The top officials of the Government of Bangladesh are frequently labelling the Rohingyas as a “burden” (UN News, 2019) which resonates with the findings of other studies (e.g., Chatham House, 2015; Miller, 2018; Fajth et al., 2019) and will likely to have detrimental impact on host community’s perception towards the Rohingyas.

## 6 Conclusion

This paper encompasses the perceptions of the host community in Cox’s Bazar and the views of Bangladeshi humanitarian professionals who are involved in supporting the Rohingyas in various capacity. Overall, the evidence presented in this paper supersede some positive impacts of hosting refugees such as productivity, local labour supply, international attention and increased external aid (as described in existing literatures such as Whitaker, 2002; Landau, 2008; Betts et al., 2014). In contrast, this study found a common narrative that delineates Rohingyas are a burden for Bangladesh. This finding is reminiscent of the pattern of hosting large number of refugees in Tanzania, Uganda, Rwanda and Jordan (see Chatham House, 2015; Ruaudel and Morrison-Métois, 2017; Miller, 2018; Fajth et al., 2019; Bjørkhaug, 2020). The length of the crisis has resulted in competition for livelihood opportunities and the lack of progress in repatriation (further exacerbated by the 2021 military coup in Myanmar) have also convoluted the situation. The host community initially felt that the Rohingyas would stay in Bangladesh for a short term. Evidently, at the start of this crisis, there has been great sympathy from the host community towards the Rohingyas. However, as the crisis got protracted and there is no significant progress in the repatriation of the Rohingyas, such perceptions has begun to change. For many host community members, prolonged stay of the Rohingyas in Bangladesh means that *their* lands, forests, and ecology have been utilised to support the Rohingyas. Furthermore, there is a growing tension due to various unlawful activities of some Rohingyas as manifested in this paper (e.g., drug peddling; prostitution; human trafficking; factional killings etc.). Restricted mobility and lack of income opportunities within the camps may have pushed the Rohingyas to pursue risky options including thefts and mugging. Some Rohingyas have also attempted to find cash-earning opportunities in the local area (e.g., as informal labourers in agriculture and other sectors), resulting in competition for limited resources and livelihood opportunities. Initially, the international community almost entirely focused on providing humanitarian support to the Rohingyas, which also caused some frustration and hostility toward this group. Despite some recent initiatives to appease the host community, our evidence suggests that the host community feel very little support is visible/available for them. Although some members of the host community feel that the Rohingya crisis has created opportunities for national and international humanitarian workers, but such opportunities are available largely for middle or upper-class graduates (for national humanitarian workers), and “outsiders” from abroad (not for the locals). Although conditions in the camps are not luxurious, there is a feeling among the host community that Rohingyas have been supported with sufficient or abundant resources where they continue to struggle to make ends meet. In other words, we observed this to be a common feeling among the host community members that the Rohingyas are being supported at *their* expense. These have been instrumental in changing the host community’s perception towards the Rohingyas. Furthermore, although we have mentioned above that we the authors observed a “festive-like mood” among the community groups and political leaders (see Section 1 for more details). While empirical evidence presented in this paper offers rich details about the host community perception in particular we feel this might be symptomatic to the views of the wider community groups. In terms of political parties, we have shown above that the ruling party views through different official/government narratives articulate that Rohingyas are a burden for the country (see Section 4 for more details). For other political parties, little is known about their stance on the displaced Rohingya refugees because current politics in Bangladesh is primarily focused on converting a “credible” election that the international community would approve as a credible one along with issues such as governance, corruption, and cost of living. Apparently, solidarity from the major political parties towards the Rohingyas has waned over time. Whether silence or inaction from the major political parties, however, contributed to host communities’ changed perception towards the Rohingyas is therefore beyond the remit and scope of this paper.

This study also exhibits that perceptions of the humanitarian professionals are not very different from the host community members. This, to an extent, could be surprising. As one might think that by closely working with the Rohingyas, they would be more sensitive and sympathetic to their plights. One might also anticipate that their training and institutional affiliations (including values at their workplace) would allow them to view the Rohingyas from a different lens. Nevertheless, based on the findings of this study, they also suggested that supporting and managing Rohingya crisis has become problematic for the host community and the country (Bangladesh). Some humanitarian professionals not only resonated with the views of social discord in terms of human trafficking and unlawful activities but also expressed their concerns for safety and security issues. In addition to the issues highlighted by the host community members, some humanitarian professionals thought ecological and environmental impacts of hosting the Rohingyas in Bangladesh have also contributed to the psyche of the host community members creating a tense situation in Cox’s Bazar. We could not be sure whether the humanitarian professionals would have had different views if they were interviewed by their institutions or international researcher(s). Nevertheless, their local knowledge by living (although in a presumably safer environment) and advanced interaction with the local communities than the foreign humanitarian professionals offer a distinctive perspective.

We acknowledge that not only do the authors’ positionalities influence the data collection of this study but also the analysis of empirical data can be seen as an interpretation that is reflexive based on the authors’ positionalities. In that vein, this can be claimed that the empirical findings add to the reflexive practices of humanitarian development scholarship that might help minimising any (un)conscious institutional or hierarchical narratives in exploring the dynamics of the relationship between the Rohingya refugees and their Bangladeshi hosts. Such reflexivity also raises an important question of whether the current approach and practice of humanitarian development (especially in the context of Rohingya crisis) can place the refugees against their hosts, especially if the crisis gets protracted and the international attention and support to the refugees wither way. We argue that such views are of significant importance in bridging the gap in existing knowledge as we do not know of any study that includes the perceptions of Bangladeshi humanitarian professionals towards the Rohingyas. Not only the host community are an important stakeholder of this crisis but also, this is important to remember that the host community members are living with the reality of sheltering and accommodating around one million Rohingyas who have been forcibly displaced from their own country. We contend that, it is imperative to look at the combination of issues (as described in this paper in explaining the reasons for host community’s recent perception toward the Rohingyas), in formulating policy strategies to ensure peaceful coexistence of the host community and the Rohingyas as well as building dignified solutions of this crisis. The evidence and arguments of this paper can make a positive impact in fulfilling the promises of an inclusive approach in managing refugee crises (in this case, the Rohingya crisis) as described in major international frameworks such as the Global Compact on Refugees and Refugee Coordination Guidance (see United Nations, 2018; UNHCR, 2019). While the host communities’ views may have been largely underrepresented or excluded in major global frameworks, we argue that the accounts of the Bangladeshi host community (as detailed in this paper) would be useful to deepen extant understanding in managing and governing refugees and displaced people as 83% of world’s displaced people are hosted in low- and middle-income countries (UNHCR, 2022).

## Data availability statement

The raw data supporting the conclusions of this article will be made available by the authors, without undue reservation.

## Ethics statement

The studies involving humans were approved by Research committee at the North South University. The studies were conducted in accordance with the local legislation and institutional requirements. The ethics committee/institutional review board waived the requirement of written informed consent for participation from the participants or the participants’ legal guardians/next of kin because oral consent were obtained as many participants were not able to read (literacy was low).

## Author contributions

PK: Conceptualization, Formal analysis, Methodology, Writing – original draft, Writing – review & editing. BS: Writing – original draft, Writing – review & editing. KA: Methodology, Writing – review & editing.

## Footnotes

^1^For more details on latest data, see https://data.unhcr.org/en/documents/details/101160 (last accessed on August 1, 2023).

^2^We attempted to conduct equal number of IDIs with the humanitarian professionals, but it was not possible due to the last-minute cancellations from three humanitarian professionals.

^3^Data was collected over 7 months (between September 2020 and March 2021) after the Rohingya influx in Bangladesh in 2017.

^4^This was at the time of data collection for this study. While we are writing the crisis now enters the sixth year and there is still no sign of Rohingya repatriation anytime soon.

^5^At times respondents felt some of these activities could be criminal activities. While the activities that were mentioned may be suspicious or not compliant to Bangladeshi laws, but we cannot determine whether those activities are criminal. Since the activities can cause significant concerns and tensions, we are using illegal and/or suspicious activities in interpreting and presenting those views.

^6^Yaba is methamphetamine drug that is “common”/“popular” in South and South East Asia.

^7^However, we have been frequently reminded by the humanitarian professional respondents that Bangladesh is not a signatory of the 1951 Refugee Convention that confines the scope of recognition of Rohingyas as Refugees, and they have been addressed as forcefully displaced Myanmar nationals (FDMNs). Therefore, they are not entitled to certain rights or privileges of Refugees apart from being supported with humanitarian aid, which is an issue of frustration among the Rohingyas, as many humanitarian professionals suggested.

^8^Some homeowners may have been benefited from this but at the same time it affected the majority who do not have a house and must rent.

^9^As of February 2023, a 72% funding gap for the Rohingyas and host community can be found here https://data.unhcr.org/en/country/bgd.
